# Effects of Ischemia on Lung Macrophages

**DOI:** 10.1371/journal.pone.0026716

**Published:** 2011-11-16

**Authors:** Aigul Moldobaeva, Nico van Rooijen, Elizabeth M. Wagner

**Affiliations:** Department of Medicine, Johns Hopkins University, Baltimore, Maryland, United States of America; Heart Center Munich, Germany

## Abstract

Angiogenesis after pulmonary ischemia is initiated by reactive O_2_ species and is dependent on CXC chemokine growth factors, and its magnitude is correlated with the number of lavaged macrophages. After complete obstruction of the left pulmonary artery in mice, the left lung is isolated from the peripheral circulation until 5–7 days later, when a new systemic vasculature invades the lung parenchyma. Consequently, this model offers a unique opportunity to study the differentiation and/or proliferation of monocyte-derived cells within the lung. In this study, we questioned whether macrophage subpopulations were differentially expressed and which subset contributed to growth factor release. We characterized the change in number of all macrophages (MHCII ^int^, CD11C+), alveolar macrophages (MHCII ^int^, CD11C+, CD11B−) and mature lung macrophages (MHCII ^int^, CD11C+, CD11B+) in left lungs from mice immediately (0 h) or 24 h after left pulmonary artery ligation (LPAL). In left lung homogenates, only lung macrophages increased 24 h after LPAL (vs. 0 h; p<0.05). No changes in proliferation were seen in any subset by PCNA expression (0 h vs. 24 h lungs). When the number of monocytic cells was reduced with clodronate liposomes, systemic blood flow to the left lung 14 days after LPAL decreased by 42% (p<0.01) compared to vehicle controls. Furthermore, when alveolar macrophages and lung macrophages were sorted and studied in vitro, only lung macrophages secreted the chemokine MIP-2α (ELISA). These data suggest that ischemic stress within the lung contributes to the differentiation of immature monocytes to lung macrophages within the first 24 h after LPAL. Lung macrophages but not alveolar macrophages increase and secrete the proangiogenic chemokine MIP-2α. Overall, an increase in the number of lung macrophages appears to be critical for neovascularization in the lung, since clodronate treatment decreased their number and attenuated functional angiogenesis.

## Introduction

Prolonged organ ischemia is profoundly injurious and results in a sequelae of cellular changes. In the ventilated lung, pulmonary ischemia does not result in the loss of cellular oxygenation but rather the cessation of perfusion-dependent stimuli including a decreased delivery of glucose/metabolic substrates, decreased endothelial shear stress, accumulation of blood borne inflammatory cells, and a relative increase in oxygen. This chronic condition in the lung promotes a series of events that culminate in reversal of the ischemic state by systemic neovascularization. Pulmonary ischemia resulting from chronic pulmonary artery obstruction has been shown to cause proliferation of the systemic circulation within and surrounding the lung (bronchial arteries and intercostal arteries) in humans [1], [2], sheep [3], pigs [4], dogs [5], and rats [6]. Using a mouse model of total left pulmonary artery ischemia, we have shown that subsequent neovascularization arises exclusively from intercostal arteries [7], is initiated by reactive oxygen species [8], is dependent on early upregulation of CXC chemokine growth factors [9], and that blockade of CXCR_2_, the G-protein coupled receptor through which these predominantly macrophage and epithelial cell-derived factors operate, results in diminished angiogenesis [10], [11]. Furthermore, our laboratory has shown a significant positive correlation between the number of monocytes/macrophages in bronchoalveolar lavage fluid and the magnitude of neovascularization [12]. Histological sections of the lung after the onset of ischemia demonstrate an abundance of macrophages with some showing signs of proliferation and others, markers of apoptosis [13]. These results are consistent with the hypothesis that monocytes/macrophages within the lung contribute to systemic neovascularization of the ischemic lung. Furthermore, these observations contribute to a growing body of literature demonstrating the importance of macrophage-derived proteins to angiogenesis [14].

Macrophages are a heterogeneous group of mononuclear phagocytic cells which display remarkable plasticity and are best defined by their function within a given tissue. However, maturation state of these cells is typically based on the expression of a variety of cell-surface markers. Normally, immature monocytes migrate randomly from blood to various organs and differentiate into tissue macrophages through the coordinated expression of numerous genes [15]. After tissue injury or during local infection, blood monocytes are recruited to a specific organ as inflammatory macrophages where further differentiation can occur [16], [17]. Normally, peripheral blood monocytes circulating through the pulmonary circulation can become sequestered within pulmonary capillaries and migrate into the interstitial and alveolar spaces where they mature into alveolar macrophages. Studies in mice demonstrated that the generation of alveolar macrophages involves the differentiation of immature blood monocytes into macrophages expressing β integrins CD11B+, CD11C+/− in the lung parenchyma, proliferative expansion of these cells, and their subsequent emigration into the alveolar space (CD11B−, CD11C+; [18]. Given our model of complete pulmonary ischemia after left pulmonary artery ligation, the fate and activity of trapped blood monocytes as well as resident macrophages is not clear. Both reactive oxygen species (ROS) and lung matrix fragments are known to stimulate macrophages [19]. Our previous work demonstrates that pulmonary ischemia results in an early increase in ROS [8] and hyaluronan fragmentation [20]. Since the mouse lung lacks an intrathoracic bronchial circulation, the ischemic lung is completely isolated from the peripheral circulation. Inflammatory macrophages that might be recruited cannot access the lung until a new systemic vascular bed is established 5–7 days after LPAL [7]. Thus, the model offers the unique opportunity to examine early monocyte differentiation in an organ without peripheral influence. In the present study, we sought to better characterize the phenotype and function of trapped monocytes/macrophages in the lung during acute pulmonary ischemia. We hypothesized that monocytes both differentiate and proliferate in response to sustained pulmonary ischemia and contribute to lung remodeling through subsequent systemic angiogenesis.

## Methods

### Animals

The use of mice followed an *in vivo* protocol approved by the Johns Hopkins Animal Care and Use Committee (Protocol number MO10M257). We studied male mice (C57Bl/6, 5–6 weeks old; Charles River Wilmington, MA) as was previously described [12], [21]. Mice were anesthetized (2% isoflurane), intubated and ventilated (120 breaths/min, 0.2 ml/breath). A left lateral thoracotomy was performed followed by ligation of the left pulmonary artery (LPAL). The left lung was harvested either immediately upon LPAL (0 h LPAL) or the thoracotomy was closed, the mouse was extubated and allowed to recover for a specified time. Sham mice were treated similarly to LPAL mice in all respects except did not undergo LPAL. For lung tissue acquisition and bronchoalveolar lavage, anesthetized mice were killed by cervical dislocation at specified times after LPAL.

### Bronchoalveolar lavage

Immediately after death, the entire right lung was ligated and the left lung was washed (3×0.3 ml of 0.2% BSA/PBS with 2 mM EDTA, 37°C). Bronchoalveolar lavage fluid was gently aspirated, the total recovered volume recorded, and total cell count (Bright Line Hemacytometer; Horsham, PA), and differential cell counts (Cytospin 4; Shandon, Pittsburgh, PA and Diff-quick staining; Dade Bering, Newark, DE) were determined where approximately 800 cells/mouse were evaluated. For flow cytometric analysis of lavaged cells, cells were labeled according to protocols applied for lung cells.

***In vitro monocyte culture and evaluation:*** Left and right lungs from naïve mice were harvested after cervical dislocation. Lungs were intubated and treated with collagenease (1 mg), minced and incubated at 37°C, then pushed through a 70 µm nylon cell strainer (BD Falcon). Erythrocytes were lysed (ACK buffer) and CD11C+ cells were collected using CD11C microbeads (Miltenyi Biotec, Auburn, CA). Cells (1–2×10^5^/well/250 µl) were plated on 96-well dishes (in DMEM supplemented with 10% FCS and antibiotics:penicillin, streptomycin, gentamicin). Cells were allowed to adhere (18 h) and were incubated with vehicle or stimuli in DMEM for 24 h. Stimuli included known macrophage stimulants H_2_O_2_ (100 µM) and low molecular weight hyaluronan (130 kDa, 250 µg/ml). Concentrations were selected based on pilot studies and previously published observations [22]. After incubation, supernatant from cultured cells was collected and MIP-2α was measured by ELISA (R&D Systems, Minneapolis, MN).

### Inflammatory cell identification by flow cytometry

Freshly extracted mouse lung tissue was minced, digested (25 min in collagenase at 37°C), and then pushed and washed through a 70 µM cell strainer. After lysing erythrocytes (ACK buffer), cells were washed and the cell pellet was collected. Nonspecific binding sites were blocked (Fc Block; BD Pharmingen, San Jose, CA), and cells were incubated with monoclonal antibodies (20 min on ice). Staining reagents for monocytes/macrophages included: APC-Alexa fluor 750-labeled anti-CD11B, PerCP Cy5.5-labeled anti-mouse CD11C, Alexa-700-labeled anti-IA/IE (MHCII), APC labeled F4/80 (Ebioscince, San Diego, CA), FITC labeled Gr-1 (BD Pharmingen, San Diego, CA) and LIVE/DEAD® Fixable Blue fluorescent reactive dye (Invitrogen, Carlsbad, CA). Mouse isotype IgG served as a negative control and only live cells were analyzed. Staining for live cells was performed after cell surface marker staining, cells were washed (PBS) and LIVE/DEAD® dye (1 µl) was added. Flow cytometry was performed with a FACSAria (Becton Dickinson, Franklin Lanes, NJ), and FlowJo software (Treestar Inc, Ashland, OR) was used to analyze results. To ensure that all samples had adequate cell numbers for monocyte characterization in both bronchoalveolar lavage and lung, pooled samples from left lungs of 6 mice were used for each experiment at each time point. Only live cells were evaluated and defined as macrophages (MHCII ^int^, CD11C+) and further subdivided as alveolar macrophages (MHCII ^int^, CD11C+, CD11B−) and lung macrophages (MHCII ^int^, CD11C+, CD11B+) [18]. In preliminary studies, the MHCII ^int^, CD11C+ cell population was predominantly F4/80+, whereas neutrophils (F4/80−, Gr-1^high^) represented less than 3% of this population. This analysis is based partially on the work of Tighe [17]. In additional experiments, alveolar macrophages (MHCII ^int^, CD11C+, CD11B−) and lung macrophages (MHCII ^int^, CD11C+, CD11B+) from lungs 0 h and 24 h after LPAL were sorted, cells were plated (as above, in vitro cells), and 18 h later, supernatants were collected for MIP-2α protein determination (ELISA; R&D Systems).

### Proliferation

Monocyte proliferation was determined by fluorescent PCNA and FACS analysis as previously described [23]. Briefly, cells from left lungs were washed (PBS), stained (LIVE/DEAD® and antibodies to MHCII, CD11C, CD11B), fixed and permeabilized using a commercially available kit (Ebioscience) for further PCNA staining, and analyzed. Flow cytometry was performed as described above.

### Monocyte/macrophage depletion with clodronate liposomes

Mice were treated with clodronate liposomes [24] 100 µl; i.p.) or control PBS liposomes (100 µl; i.p.) 24 h before LPAL. Preliminary studies demonstrated that this treatment resulted in a 53% reduction in tissue macrophages 24 h after LPAL. Consequently, additional liposome treatments (100 µl; i.p. and 40 µl intratracheal aspiration) were given 1, 5 and 9 days after LPAL (long treatment) or in another series of mice, only 1 day after LPAL (short treatment). Angiogenesis was determined 14 days after LPAL.

### Angiogenesis assessment

After mice were anesthetized and intubated, the extent of neovascularization was determined by measuring systemic blood flow to the left lung 14 days after LPAL using fluorescent microspheres (Invitrogen, Eugene, OR). The carotid artery was cannulated and 360,000 microspheres (10 µm) were infused. Mice were sacrificed by exsanguination and the left lung was excised. Microspheres lodged in the left lung were quantified after tissue digestion and fluorescent dye extraction, and the number calculated from a standard curve. Data are presented as the percent total microspheres delivered, ie, % cardiac output.

### Statistical analysis

Data are presented as the means ± standard errors. ANOVA was used to analyze changes in the number of lavaged monocyte/macrophages. Student's t-test was used to compare macrophage number 0 h vs 24 h, changes in blood flow and absolute chemokine release and the Newman-Keuls Multiple Comparison test was used for evaluation of cell number after clodronate treatment. Percent change data were analyzed using Wilcoxon Signed Rank test. A p value<0.05 was accepted at statistically significant.

## Results

### In vivo inflammatory cell identification

**Figure 1:**
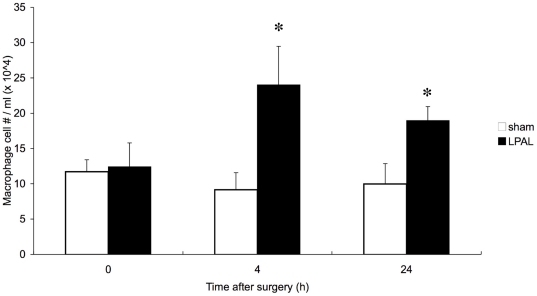
Average early changes in macrophage numbers in bronchoalveolar lavage fluid in sham operated mice and mice after LPAL (n = 3–5 mice/group). Total cell counts paralleled the changes in macrophage number (* indicates p<0.05 sham vs LPAL).

The early time course of the increase in macrophage numbers in bronchoalveolar fluid was confirmed in the present series. Figure 1 shows the number of lavaged monocytes/macrophages in mice immediately after LPAL (0 h) and early (4 h and 24 h) after LPAL compared to mice undergoing sham surgery. As previously shown [12], mice undergoing sham surgery showed no change in the number of lavaged macrophages, however, a substantial increase in the number of monocytes/macrophages was observed 4 h and 24 h after LPAL (n = 3–5 mice/group; p<0.05 compared to sham). These increases paralleled the total number of lavaged cells (2.6- and 2-fold increase vs sham, 4 and 24 h after LPAL; p<0.05). Recovered volumes did not differ among groups and averaged 0.7 ml for all mice (∼80% recovery). Since the 0 h values did not differ from sham operated mice at any time point, in subsequent experiments the 0 h samples served as controls.

### In vitro macrophage responsiveness

**Figure 2:**
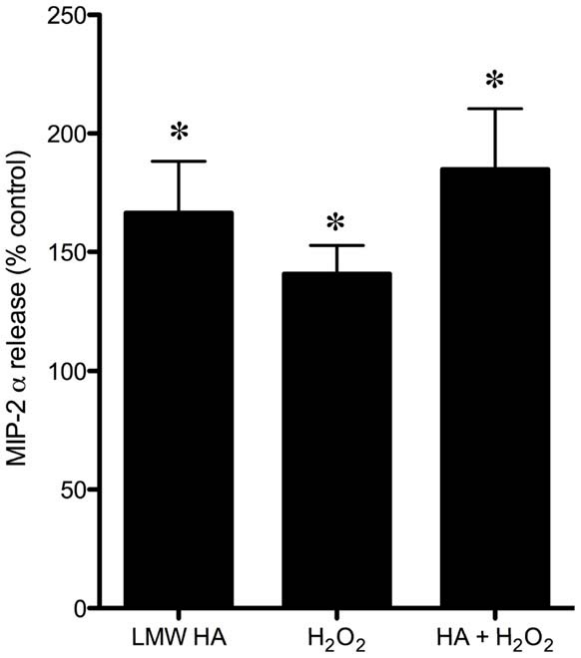
Changes in MIP-2α secretion in isolated CD11C+ macrophages exposed to low molecular weight hyaluronan (LMW HA) and H_2_O_2_. LMW HA and H_2_O_2_ were equally effective at eliciting a significant increase in MIP-2α release compared to vehicle control treatment in CD11C+ cells (p<0.05). The combined treatments did not induce an additive effect.

To explore one mechanism by which lung macrophages contribute to systemic angiogenesis, we compared the ability of naïve CD11C+ cells to secrete MIP-2α when stimulated with putative agonists generated by the ischemic lung. MIP-2α secretion averaged 491±147 pg/ml in CD11C+ cells in vehicle-treated control conditions. Figure 2 shows the percent changes of MIP-2α secretion from vehicle when CD11C+ cells were exposed to low molecular weight hyaluronan (LMW HA) and H_2_O_2_. LMW HA and H_2_O_2_ were equally effective at eliciting a significant increase in MIP-2α release compared to vehicle control treatment in CD11C+ cells (p<0.05). The combined treatments did not induce an additive effect.

### Macrophage phenotype

**Figure 3:**
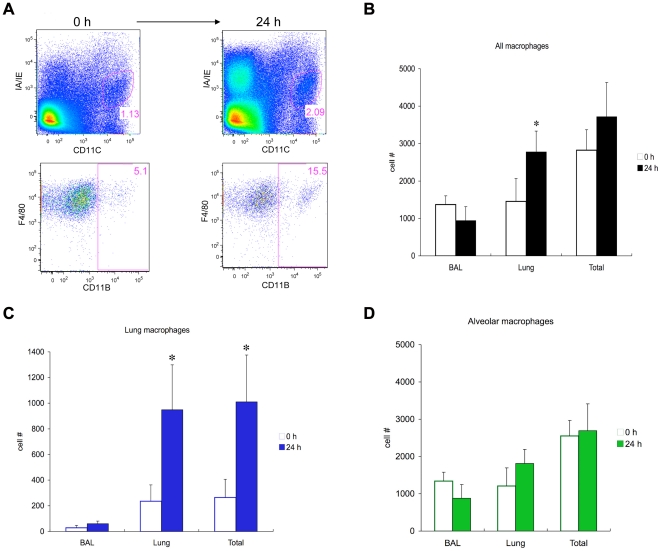
Changes in macrophage numbers in dissociated left lung tissues after LPAL. **3A:** Dot plots of dissociated lung 0 h and 24 h after LPAL. The selected region of IA/IE (MHCII)^int^, CD11C+ cells (1.13% of all live cells) increased 24 h after LPAL. As shown in the lower plots, these cells were F4/80+, and CD11B+ cells increased 24 h after LPAL to 15.5%. **3B:** Change in cell numbers of all macrophages (MHCII^int^, CD11C+) in BAL (bronchoalveolar lavage), dissociated lung, and their sum total, 0 h and 24 h after LPAL (n = 4 experiments/group; * indicates p<0.02). **3C:** Mature lung macrophages (MHCII^int^, CD11C+, CD11B+; * indicates p<0.05). **3D:** Alveolar macrophages (MHCII^int^, CD11C+, CD11B−).

**Figure 4:**
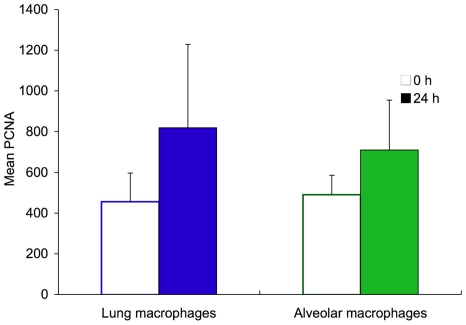
No differences were seen in mean PCNA fluorescence between lung macrophages and alveolar macrophages in lung homogenate samples immediately after LPAL (0 h). Although both subpopulations showed slight increases in proliferation 24 h after LPAL, these changes did not reach statistical significance.

To further evaluate in vivo differentiation and compartmental changes in cell type, we examined changes in all macrophages (MHCII ^int^, CD11C+), alveolar macrophages (MHCII ^int^, CD11C+, CD11B−) and lung macrophages (MHCII ^int^, CD11C+, CD11B+) [18], 24 h after LPAL compared to 0 h, separately in dissociated lung and bronchoalveolar lavage fluid. Figure 3A shows examples of dot plots of dissociated lung 0 h and 24 h after LPAL. An increase in MHCII ^int^, CD11C+ cells is observed. These cells were predominantly F4/80+ and results demonstrated an increase from 0 h to 24 h, in the number of cells that were CD11B+. Actual number of cells was determined (n = 4 experiments/time point). Figure 3B shows the cell numbers in BAL, lung, and the total number of cells for all macrophages (MHCII ^int^, CD11C+). Twenty-four h after LPAL, a significant increase in macrophages was observed compared to the cell number seen initially at 0 h (p<0.02). This change represented an average 91% increase in cell number. Changes in phenotype were more obvious when examining specifically lung macrophages (MHCII ^int^, CD11C+, CD11B+) seen in Figure 3C. A significant increase in total (average 280% increase; p<0.05) and lung homogenate lung macrophages (average 300% increase; p<0.05) and a trend toward a change was observed in lung macrophages in BAL (average 110% increase; p = 0.06). Surprisingly, no changes were seen in populations of alveolar macrophages (MHCII^int^, CD11C+, CD11B−) from BAL, lung or total number (Figure 3D). When the two macrophage subgroups were analyzed for proliferative capacity, there were no differences in mean PCNA fluorescence between lung macrophages and alveolar macrophages in lung homogenate samples immediately after LPAL (0 h; Figure 4). Although both showed slight increases in proliferation 24 h after LPAL, these changes did not reach statistical significance.

**Figure 5:**
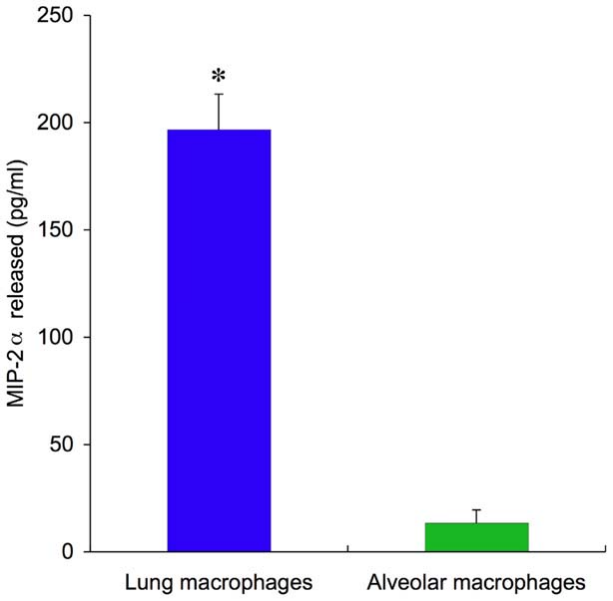
Sorted mature lung macrophages (MHCII ^int^, CD11C+, CD11B+) and alveolar macrophages (MHCII ^int^, CD11C+, CD11B−) obtained from naïve lungs or lungs after LPAL were studied after 18 h in culture. Supernatants evaluated for MIP-2α showed spontaneous release only in mature lung macrophages (* indicates p<0.0001, n = 12 lungs).

In separate experiments, sorted lung macrophages (MHCII ^int^, CD11C+, CD11B+) and alveolar macrophages (MHCII ^int^, CD11C+, CD11B−) obtained from naïve lungs or lungs after LPAL were studied after 18 h in culture. Supernatants evaluated for MIP-2α showed spontaneous release of the CXC chemokine only in mature lung macrophages from both naïve and LPAL lungs (p<0.0001, n = 12 lungs, Figure 5).

### Angiogenesis assessment after monocyte/macrophage depletion

**Figure 6:**
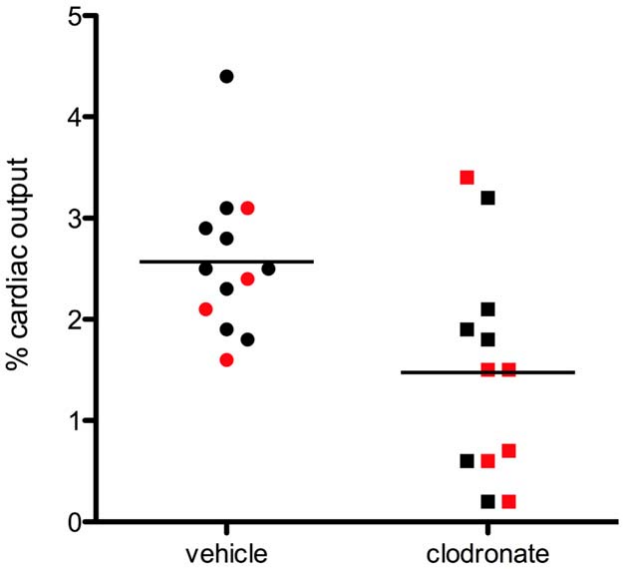
Change in angiogenic blood flow to the left lung 14 days after LPAL in vehicle control mice compared to mice treated with clodronate liposomes (each symbol represents one mouse; red symbols = short treatment, black symbols = long treatment). Mice treated with PBS-liposomes showed a typical systemic blood flow to the left lung compared with previously published values [3]. Mice treated with clodronate liposomes, known to reduce the number of monocytic cells, showed a substantial reduction in systemic perfusion (* indicates p<0.006).

The overall importance of cells of monocytic lineage to the process of angiogenesis was assessed after treatment of mice with clodronate liposomes. A long treatment before and throughout the 14 day period after LPAL with clodronate liposomes (n = 6) resulted in a 41% decrease in blood flow to the left lung compared to treatment with PBS liposomes (n = 9; p<0.05). Interestingly, 2 clodronate treatments (short treatment), only 24 h before and 24 h after LPAL resulted in a similar average 44% decrement in angiogenesis as assessed by neovascular perfusion (n = 6) and this value was not different from the results of the long multiple treatment course. Consequently, the results of the two different treatment schedules were combined and the results shown in Figure 6 with individual data points representing individual mice (red symbols: short treatment, black symbols: long treatment). Blood flow to the left lung averaged 2.6±0.2% in PBS liposome treated mice (n = 13), a value similar to what has been seen previously in wild type, untreated mice after LPAL [12]. Treatment with clodronate liposomes (n = 12 mice) resulted in an average blood flow of 1.5±0.3%, which was significantly different from vehicle-treated controls (p = 0.006). These results suggest the essential nature of monocyte- derived cells for systemic neovascularization of the lung.

**Figure 7:**
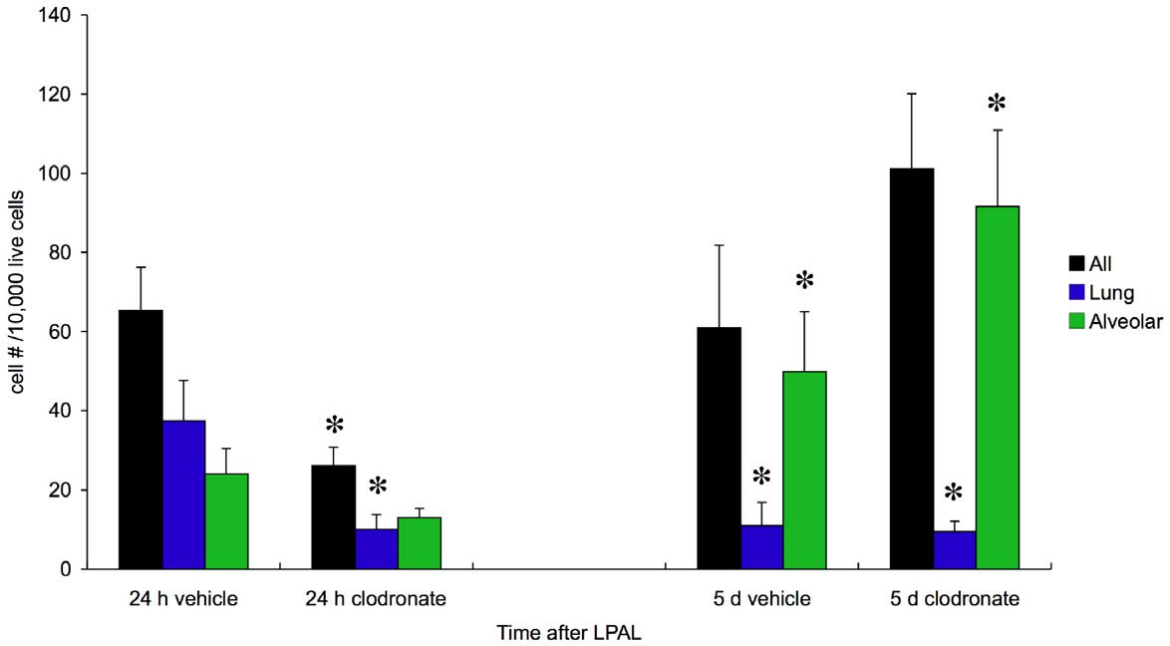
Changes in macrophage subtype in mice treated with clodronate liposomes. The effects of the short liposome treatment were assessed 24 h and 5 d after LPAL (cell #/10,000 live cells). Over the 4 groups studied (24 h and 5 d, PBS vehicle liposomes and clodronate liposomes) there were significant changes in all macrophages (p = 0.005), mature lung macrophages (p = 0.008), and alveolar macrophages (p<0.0001). Clodronate treatment (n = 9 mice) resulted in a significant decrease in the total number of macrophages by 24 h compared with PBS liposome treatment (n = 12 mice). However, by 5 d after LPAL in both vehicle (n = 4 mice) and clodronate-treated mice (n = 6 mice), the number of all macrophages within the lung was not statistically different from the 24 h vehicle level. Mature lung macrophages were significantly reduced in all groups compared to 24 h vehicle control level. The number of alveolar macrophages was not significantly altered by clodronate treatment 24 h after LPAL, however increased in both vehicle and clodronate groups by 5 d. * indicates p<0.05 compared to 24 h vehicle.

An additional series of mice was studied to confirm the changes in macrophage subtype in mice treated with clodronate liposomes (short treatment) and PBS vehicle controls. Because cells from BAL constituted a small fraction of the total number of cells (Figure 3B–3D), we extracted cells from dissociated left lungs without bronchoalveolar lavage. The effects of treatment were assessed 24 h and 5 d after LPAL and results are presented in Figure 7 (cell #/10,000 live cells). Over the 4 groups studied (24 h and 5 d, PBS vehicle liposomes and clodronate liposomes) there were significant changes in all macrophages (p = 0.005), mature lung macrophages (p = 0.008), and alveolar macrophages (p<0.0001). Clodronate treatment (n = 9 mice) resulted in a significant decrease in the total number of macrophages by 24 h compared to PBS liposome treatment (n = 12 mice; p<0.05). However, by 5 d after LPAL in both vehicle (n = 4 mice) and clodronate-treated mice (n = 6 mice), the number of all macrophages within the lung was not statistically different from the 24 h vehicle level. Mature lung macrophages were significantly reduced in all groups compared to 24 h vehicle control level (p<0.05). The number of alveolar macrophages was not significantly altered by clodronate treatment 24 h after LPAL, however increased in both vehicle and clodronate groups by 5 d (p<0.05).

## Discussion

Macrophages have long been implicated as central participants in the process of angiogenesis [25], [26]. Both as a source of endothelial cell growth factors [14], as well as matrix metalloproteinases essential to the invasion of new vessels [27], macrophages have been shown to be abundant at the sites of neovascularization and critical to the process. Previous work from our laboratory showed a significant positive correlation between the number of macrophages in the bronchoalveolar lavage fluid of the left lung after complete pulmonary ischemia and the extent of systemic angiogenesis [12]. Furthermore, macrophage-derived growth factors, the CXC chemokines, are upregulated in the ischemic lung [9] and are essential to the process of neovascularization [10], [11]. In the present study, we sought to better characterize the trapped monocytes/macrophages in the lung early after the onset of pulmonary ischemia. Although, previous studies have shown monocyte differentiation in lung injury models and the recruitment of specific macrophage subtypes, our model offered an opportunity to study changes in monocyte phenotype in a closed, in vivo environment where no new cells could be recruited until 5–7 days after LPAL when new vessels were formed. We hypothesized that monocytes both differentiate and proliferate in response to sustained pulmonary ischemia and contribute to lung remodeling through subsequent systemic angiogenesis. Our experimental results demonstrate an increase in the number of mature lung macrophages early after the onset of ischemia and suggest an essential nature of these cells for angiogenesis.

In the present study, we first confirmed our previous observations demonstrating a significant increase in the number of monocytes/macrophages that could be washed from airspaces of the left lung 4 h and 24 h after LPAL compared to mice undergoing sham surgery. At these early time points after the onset of complete left lung ischemia, the source of the increase in cells in the alveolar compartment could be only from within the lung (interstitium, vasculature and/or proliferation). This is because the mouse has no intrathoracic bronchial vasculature and a new systemic angiogenic bed is not formed until 5 days after LPAL [7]. The increase in macrophage number was comparable to what was previously reported [12].

Our next experiment confirmed that lung macrophages could contribute to growth factor release in this model. Since we have shown that CXC chemokines are essential to the process of neovascularization [10], [11], we sought to first confirm that macrophages are a relevant source of these growth factors. We challenged CD11C+ cells with hyaluronan fragments and H_2_O_2_, since we have shown previously that ROS initiate the cascade of events leading to systemic angiogenesis of the lung [8] and hyaluronan fragmentation also contributes to neovascularization [20]. We showed that these ischemia-induced agonists caused a significant increase in the secretion of the MIP-2α, one of the CXC chemokines, from CD11C+ cells. Others have shown these agonists to elicit the release of several additional cytokines [19]. Based on this in vitro study, we speculate that the increase in macrophages within the lung during ischemia, contributes to the chemokine burden within the left lung shown previously for this model and important for angiogenesis [9], [10], [11].

We subsequently questioned whether the increase in lavaged macrophages observed originally, was due only to a movement of macrophages into the alveolar compartment or whether the increase reflected proliferation or differentiation in the lung. Consequently, the 24 h time point was selected for a more complete characterization of surface marker expression of monocytes/macrophages in alveolar versus lung compartments and their total. When evaluating all live macrophages using flow cytometry, we saw significant increases only in samples of dissociated lung tissue (Figure 3A). We can only suggest that the reason we did not see a change in BAL cell numbers was related to more rigid criteria used to identify this distinct population of macrophages based on specific surface markers as well as the potential differences due to processing cells for the two different evaluations. Furthermore, the increase in macrophages in the lung compartment was due entirely to the increase in mature lung macrophages expressing MHCII^int^, CD11C+, CD11B+ (Figure 3B) and not to changes in alveolar macrophages (MHCII^int^, CD11C+, CD11B−; Figure 3C). We apply the nomenclature used by Landsman and colleagues who showed that, based on surface marker expression, monocytes normally differentiate within the lung from an immature state to mature lung macrophages and finally to alveolar macrophages [18]. Because the left lung in this model excludes peripheral blood macrophages and no additional cells can be newly recruited to the lung within the first 24 h after LPAL, this classification is appropriate. Furthermore, no differences were seen in the proliferation between these two cell types (Figure 4). Thus, we conclude from this phenotyping experiment, that there was differentiation of immature monocytes in the lung to a more mature state (MHCII^int^, CD11C+, CD11B+) due to stimuli related to the ischemic stress. This was somewhat reflected in the BAL which showed a trend toward increased lung macrophages 24 h after LPAL (p = 0.06).

On further examination of the two macrophage subpopulations, it was the mature lung macrophages (MHCII^int^, CD11C+, CD11B+) and not alveolar macrophages that, after sorting and culturing in vitro, spontaneously released the CXC chemokine MIP-2α (Figure 5). The flow cytometric analysis used in the present study was partially modeled after the work of Tighe and colleagues [17]. Interestingly, these investigators demonstrated that the macrophage subpopulation that they termed exudative macrophages (MHCII^int^, CD11C+, CD11B+) were recruited to the lung after bleomycin injury. Furthermore, it was this group of cells that when stimulated in vitro with LPS and hyaluronan fragments, produced MIP-2α. Despite the inability to recruit circulating inflammatory cells in the LPAL injury model, it appears that these cell populations may be the same and may be activated by similar stimuli.

No changes in proliferation were observed in macrophage subpopulations 24 h after LPAL. Our laboratory previously reported an increase in the PCNA positive cells in the lung parenchyma after LPAL by immunohistochemistry [13]. However, this change was not seen until 3 days after LPAL and included macrophages as well as alveolar type II cells and bronchial epithelial cells. The present work is consistent with prior results.

The importance of mature lung macrophages to the process of angiogenesis was suggested by treating mice with clodronate liposomes which deplete cells of monocytic lineage [24]. We made functional measurements of angiogenesis determined by systemic perfusion to the left lung 14 d after LPAL. Treatment before and throughout the 14 d period after LPAL with clodronate liposomes (long treatment) resulted in a 41% decrease in blood flow to the left lung compared to treatment with PBS liposomes. Blood flow to the left lung in PBS liposome treated mice resulted in similar values to what has been seen previously in wild type, untreated mice after LPAL [12]. Interestingly, in another group of mice treated with clodronate liposomes, only 24 h before and 24 h after LPAL (short treatment), showed a similar 44% decrement in angiogenesis. In additional mice, we confirmed the change in number and macrophage phenotype in mice treated with clodronate (short treatment; Figure 7). Clodronate liposome treatment resulted in a significant decrease in the total number of macrophages by 24 h compared with PBS liposome treatment. However, by 5 d after LPAL in both vehicle and clodronate-treated mice, the number of all macrophages within the lung was not different from the 24 h vehicle level. Mature lung macrophages were reduced 24 h after LPAL in the clodronate treated mice and remained decreased at 5 d LPAL in both vehicle and clodronate treated mice. The results of macrophage phenotype determination (Figure 7) and angiogenesis with long and short clodronate treatments (Figure 6) highlight the importance of lung macrophages early in the process of neovascularization. The longer and shorter treatments resulted in similar effects on angiogenesis measured 14 d after LPAL yet the shorter clodronate treatment was no longer effective at keeping the level of all macrophages suppressed after 5 d LPAL. Additionally, mature lung macrophages, the primary chemokine-producing cells (Figure 2) were significantly decreased after 5 d LPAL yet did not differ between vehicle and clodronate groups. These results further suggest the importance of mature lung macrophages as chemokine growth factor producing cells very early after LPAL (<5 d). This interpretation is consistent with our previous work that demonstrated chemokine expression and protein is an early event (0–3 days; [9]. Although clodronate may have had a direct effect on blood vessel formation, we believe these results show a strong association of the early differentiation of monocytes to mature lung macrophages with the process that leads to systemic neovascularization of the lung.

Several recent review articles highlight the heterogeneity and plasticity of cells of monocyte lineage [28], [29]. In the present study, we have taken advantage of the mouse lung which lacks an intrathoracic bronchial vasculature and observed changes in macrophage phenotype after LPAL that underscore monocyte plasticity. In summary, our results demonstrate that ischemic stress within the lung contributes to the differentiation of immature monocytes to mature lung macrophages within the first 24 h after LPAL. Lung macrophages but not alveolar macrophages increase in number early after ischemia and secrete the proangiogenic chemokine MIP-2α. Overall, an increase in the number of lung macrophages appears critical for the process of neovascularization in the lung, since clodronate treatment decreased their number and attenuated functional angiogenesis.
